# Engineering a disulfide-gated switch in streptavidin enables reversible binding without sacrificing binding affinity

**DOI:** 10.1038/s41598-020-69357-5

**Published:** 2020-07-27

**Authors:** Jesse M. Marangoni, Sau-Ching Wu, Dawson Fogen, Sui-Lam Wong, Kenneth K. S. Ng

**Affiliations:** 1 Department of Biological Sciences, University of Calgary, Calgary, AB T2N 1N4 Canada; 2 Present Address: Department of Molecular Biosciences and Bioengineering, University of Hawaii, Honolulu, HI 96822 USA

**Keywords:** Biochemistry, Proteins, Biological techniques, Molecular engineering, Protein design

## Abstract

Although high affinity binding between streptavidin and biotin is widely exploited, the accompanying low rate of dissociation prevents its use in many applications where rapid ligand release is also required. To combine extremely tight and reversible binding, we have introduced disulfide bonds into opposite sides of a flexible loop critical for biotin binding, creating streptavidin muteins (M88 and M112) with novel disulfide-switchable binding properties. Crystal structures reveal how each disulfide exerts opposing effects on structure and function. Whereas the disulfide in M112 disrupts the closed conformation to increase *k*_*off*_, the disulfide in M88 stabilizes the closed conformation, decreasing *k*_*off*_ 260-fold relative to streptavidin. The simple and efficient reduction of this disulfide increases *k*_*off*_ 19,000-fold, thus creating a reversible redox-dependent switch with 70-fold faster dissociation kinetics than streptavidin. The facile control of disulfide formation in M88 will enable the development of many new applications requiring high affinity and reversible binding.

## Introduction

Streptavidin is a 66-kDa, homotetrameric biotin-binding protein first isolated from the bacterium *Streptomyces avidinii*^1^. The streptavidin–biotin complex has an equilibrium dissociation constant of 4 × 10^–14^ M, one of the strongest non-covalent interactions found in nature^2^. An 8-stranded anti-parallel β-barrel forms the core structure of each streptavidin protomer, inside of which three key Trp residues (residues 79, 92 and 108) form a hydrophobic binding pocket that interacts with the largely hydrophobic tetrahydrothiophene ring and hydrocarbon portion of the valeric acid moiety of biotin^3,4^. Trp-120, also forms part of this binding pocket, but is contributed by another subunit of the streptavidin tetramer. In addition to the hydrophobic pocket, the biotin ureido oxygen participates in an intricate hydrogen-bonding network mediated by Asn-23, Ser-27 and Tyr-43^5–7^.

The loop connecting β-strands three and four, which is often called “loop 3–4” (residues 45–52), also makes important contributions to biotin binding. When biotin is absent, this loop appears to be highly flexible and dynamically disordered, but it closes upon ligand binding, further burying biotin in the pocket. Closure of the loop promotes the formation of hydrophobic contacts between the valeric acid acyl chain of biotin and the hydrophobic side chain of Val-47 and the alpha carbon of Gly-48, while the carboxylate group of the valeric acid moiety accepts hydrogen bonds from the backbone amide group of Asn-49. Additionally, the hydroxyl group of Ser-45 forms a hydrogen bond with one of biotin’s ureido nitrogens. The contribution of this loop to binding affinity was elegantly demonstrated in circularly permuted streptavidin, where the removal of loop 3–4 resulted in a decrease in binding affinity of approximately six orders of magnitude (K_d_ = 1 × 10^−7^ M)^8^.

Since most biomolecules can be readily biotinylated using commercially available reagents targeting amino, thiol and carboxylate functional groups, the uniquely tight and selective streptavidin–biotin interaction has been widely exploited for biological research, biotechnology and biomedical applications^9,10^. However, streptavidin’s high affinity binding with biotin is weakened when biotin is conjugated to other molecules, especially larger molecules that are more sensitive to shearing, mechanical forces. For example, streptavidin coated beads in a laminar flow chamber roll across the biotinylated surface rather than stably binding to it^11^, and the apparent equilibrium dissociation constant for biotinylated DNA and streptavidin-coated polystyrene particles increases with both DNA length and particle size^12^. To address some of these limitations, mutations were introduced to increase the rigidity of loop 3–4, giving rise to the traptavidin mutant which shows a tenfold decrease in the rate of dissociation, partially mitigating the effects of mechanical forces^13–15^.

Conversely, while high-affinity binding is desirable for many applications, the slow rate of dissociation for biotin from wild-type streptavidin can also be a major limitation. High temperatures, extreme pH and chaotropic salts can weaken binding, but these strongly denaturing conditions often lead to undesirable negative effects on target molecules and cells, as well as streptavidin itself, especially after multiple rounds of treatment. Because of these limitations, many mutants^16^ and biotin analogues (e.g., iminobiotin^17^ and desthiobiotin^18^) have been developed to reduce the strength of binding and to increase the rate of dissociation. However, such strategies are not viable for many applications where high affinity binding is required.

Previous attempts to address these potential limitations have only been partially successful. One approach relies on engineering intermolecular disulfide bonds between biotinylated peptide tags or streptavidin binding peptides and streptavidin^19,20^. Although these intermolecular disulfide bonds successfully combine the high strength of covalent interactions and redox-dependent reversible binding, they can only be used for carefully engineered proteins and peptides, restricting their utility and increasing costs and complexity. A second approach relies on introducing mutations that disrupt some of the binding interactions stabilizing the closed conformation of the biotin-streptavidin complex, leading to a weakening of binding and increase in rate of dissociation^21,22^. To create a more general solution to the central problem of increasing rates of dissociation without compromising the desirability of extremely high binding affinity, we report for the first time streptavidin mutants in which the reversible formation and breakage of a strategically located intramolecular disulfide bond acts as a molecular switch converting between a high affinity/low rate of dissociation state and a low affinity/high rate of dissociation state. Our approach exploits the effects of loop 3–4 dynamics on biotin binding and release, especially the observation that the closed conformation presents a steric barrier to biotin escape. We hypothesized that introducing a strategically placed disulfide bond could greatly stabilize the closed conformation of loop 3–4 while allowing for the open conformation when the disulfide bond was reduced. As a result, this engineered form of streptavidin could be reversibly converted between high-affinity/low rate of dissociation and low-affinity/high rate of dissociation states by controlling the formation and breakage of the engineered disulfide bond using mild oxidizing and reducing agents.

To test this hypothesis, we created two mutants (designated M88 and M112) of streptavidin, each with a single disulfide bond located on opposite sides of loop 3–4. Each disulfide bond exerts opposing effects on biotin dissociation kinetics, with the M88 (N49C/A86C) mutein reducing the rate of biotin dissociation in the oxidized form and M112 (G26C/A46C) mutein surprisingly increasing the rate of biotin dissociation. To explain these differing results, we determined the three-dimensional structures of the oxidized states of each protein using X-ray crystallography and also discovered different effects on protein thermostability that are consistent with the structural changes observed in the disulfide-bonded forms. For both muteins, the formation and breakage of the engineered disulfide bond had dramatic effects on biotin-binding affinity. Although M112 does not appear to be a promising candidate for most practical applications due to its slow rate of disulfide bond formation, M88 has many attractive properties: the M88 disulfide bond forms rapidly, decreases the rate of biotin dissociation 260-fold in its oxidized form and increases the rate of dissociation 70-fold relative to wild-type streptavidin when the disulfide bond is reduced (at 21 °C). Even though the dissociation rate for the reduced form, with a half-life of ~ 1 day at 21 °C, is still too slow for many practical applications, the dramatic 19,000-fold change in rates of dissociation between the reduced and oxidized states indicates that this mutein provides a very promising starting platform for further engineering to allow both high-strength and reversible binding in a single protein.

## Results and discussion

### Rationale for the design of novel disulfide bonds

**Figure 1:**
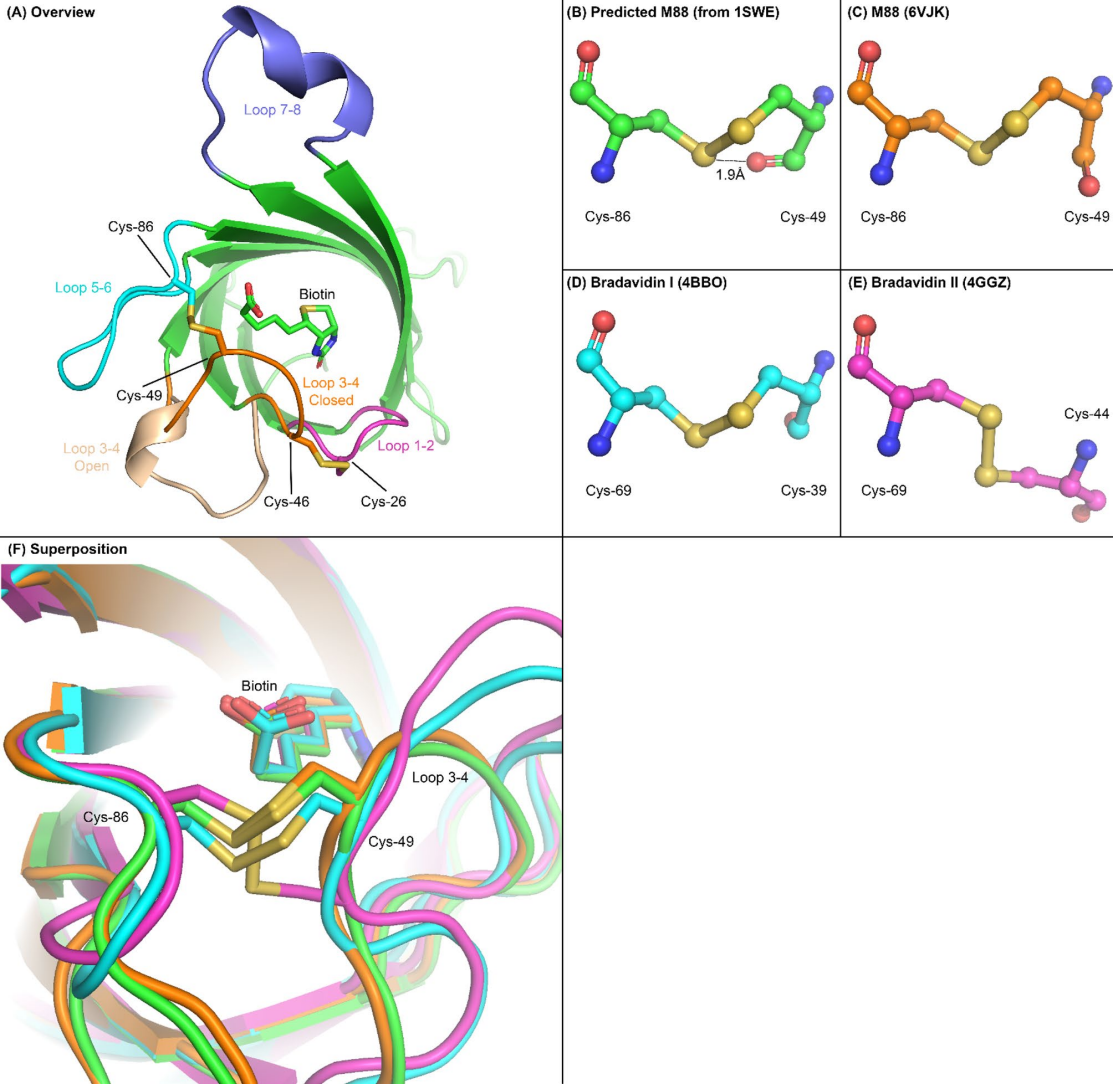
Overview structure of a single protomer of streptavidin and detail of the M88 disulfide bond. (**A**) The biotin binding site of a streptavidin protomer (Chain A from 1SWE) with the Cys mutations predicted by Disulfide by Design 2.0. Also highlighted are the β-strand connecting loops on the biotin binding side of the monomer. The Cys-26/Cys-46 pair in M112 connects the N-terminal side of loop 3–4 (residues 45–53) to loop 1–2 (residues 23–27), whereas the Cys-49/Cys-86 pair in M88 connects the C-terminal side of loop 3–4 with loop 5–6 (residues 79–87). The open conformation of loop 3–4 from apo-streptavidin (1SWC) has been superimposed on the structure for comparison with the closed conformation. Loop 7–8 (residues 113–122), while not contributing to intrasubunit ligand binding, makes contact with the binding pocket of an adjacent subunit in the tetramer. (**B**) Close-up of Cys mutations predicted by Disulfide by Design 2.0 in wild-type streptavidin. Also shown is the distance between the sulfur of Cys-49 and the oxygen of the backbone carbonyl group, indicating a steric clash. (**C**) Disulfide bond observed in the M88 crystal structure. (**D**) Disulfide bonds of Bradavidin I^52^ and (**E**) Bradavidin II^27^, two streptavidin homologs with naturally occurring disulfide bonds. Most natural disulfide bonds, with the exception of Bradavidin I, are similar in geometry to Bradavidin II, which was chosen as a representative example. (**F**) Alpha-carbon traces shown as tubes and disulfide bonds and biotin shown as sticks for the four structures shown in panels **B**–**E**. Colouring is the same as in panels B-E.

Disulfide by Design 2.0^23^ was used to predict suitable locations for introducing two pairs of cysteine residues in loop 3–4 of the biotin-bound streptavidin crystal structure 1SWE^7^ that are expected to allow for the formation of disulfide bonds with favourable geometry when loop 3–4 is in the closed conformation. This analysis indicated that disulfide bonds with suitable geometry could be formed by the G26C and A46C mutations in the mutein designated M112, and by the N49C and A86C mutations in the mutein designated M88 (Fig. 1A). Disulfide by Design also predicted that the torsion angles of the disulfide bond formed in M88 would be closer to the angles seen in natural disulfide bonds and thus would be expected to form more favourably.

It is notable that several streptavidin homologs with naturally occurring disulfide bonds in loop 3–4 have been previously reported^24–28^. Even though these homologs only share 20–30% sequence identity with streptavidin, the β-barrel fold and many elements of the tertiary structure are highly conserved. In all cases, these natural disulfide bonds occur in the C-terminal side of loop 3–4 (equivalent to residues 49–52 in wild-type streptavidin). Superposition of these structures with the model of streptavidin containing the predicted disulfide bond in M88 shows clear similarity with the crystal structure of bradavidin I (Fig. 1B,D–F). Furthermore, the crystal structure of a circularly permuted wild-type streptavidin mutant in which the protein termini are shifted to the middle of loop 3–4 (between residues 48 and 49), showed that residues 49–52 are found in the open conformation even when biotin is bound^29^, suggesting that constraining the C-terminal side of loop 3–4 in the closed conformation may have a dramatic effect on inhibiting biotin dissociation. These observations and the invariable location of natural disulfide bonds in the C-terminal half of loop 3–4 are consistent with the expectation that forming a disulfide bond between newly introduced cysteine residues at positions 49 and 86 in M88 could have a strongly stabilizing effect on biotin binding.

### The crystal structure of M88 confirms the formation of a disulfide bond in the biotin-bound closed conformation of loop 3–4

The oxidized form of M88 was crystallized in the presence of biotin, yielding well-diffracting crystals belonging to space group P2_1_2_1_2_1_ and showing a novel crystal packing arrangement of three tetramers in the asymmetric unit that differs from the packing arrangements seen in previously published structures of streptavidin or mutants (Table 1). With a root-mean square deviation of 0.38 Å over the 118 alpha-carbon atoms common to M88 chain A and 1SWE chain A, the core structure of M88 is quite similar to wild-type streptavidin, with no particularly notable differences. The disulfide bond formed in M88 is also nearly identical in geometry to that predicted by Disulfide by Design 2.0 and that of the natural homolog bradavidin I (Fig. 1B,C,D,F). The most important difference between the experimentally determined crystal structure and the model is a small rotation of the Cys-49 carbonyl group that moves it away from the newly introduced disulfide bond to resolve a steric clash, but with no major changes to the structure of either loop 3–4 or loop 5–6.

**Table 1:** Crystallographic statistics.

Crystal	M88 (oxidized) + biotin	M112 (oxidized) + biotin
PDB code	6VJK	6VJL
Space group	P2_1_2_1_2_1_	I4_1_22
Unit cell dimensions a, b, c (Å)	60.260 79.549 281.549	57.772 57.772 184.419
Wavelength (Å)	0.9795	0.9795
Resolution (Å)^a^	50–1.60 (1.69–1.60)	40–1.30 (1.33–1.30)
R_sym_^b^	0.133 (0.747)	0.073 (1.008)
CC_1/2_	0.994 (0.814)	1.00 (0.577)
I/σ	13.0 (5.4)	19.6 (1.49)
Completeness (%)	98.7 (98.8)	98.9 (94.1)
Redundancy	7.8 (8.1)	11.0 (5.6)
**Refinement**		
Resolution (Å)	1.60	1.30
Unique reflections	176,700	38,946
R_work_^c^/R_free_^d^	0.184 / 0.215	0.190 / 0.201
Total No. atoms	12,685	1,090
Protein atoms	10,826	918
Ligand atoms	192	16
Water atoms	1667	156
Avg B-factors (protein)	19.4	17.7
Avg B-factors (ligand)	14.1	12.1
Avg B-factors (water)	31.8	33.4
**r.m.s.d. from ideal geometry**		
Bond lengths (Ǻ)	0.007	0.007
Bond angles (°)	1.324	1.336

^a^Values from the outermost resolution shell are given in parentheses.

^b^R_sym_ = ∑_i_ | I_i_—< I >| / ∑i I_i_ where I_i_ is the ith integrated intensity of a given reflection and < I > is the weighted mean of all measurements of I.

^c^R_work_ = ∑||***F***_***o***_|—|***F***_***c***_|| / ∑|***F***_***o***_| for 95% of reflection data used in refinement.

^d^R_free_ = ∑||***F***_***o***_|—|***F***_***c***_|| / ∑|***F***_***o***_| for 5% of reflection data excluded from refinement.

### The M88 disulfide forms rapidly with the addition of exogenous oxidizing agents

**Figure 2:**
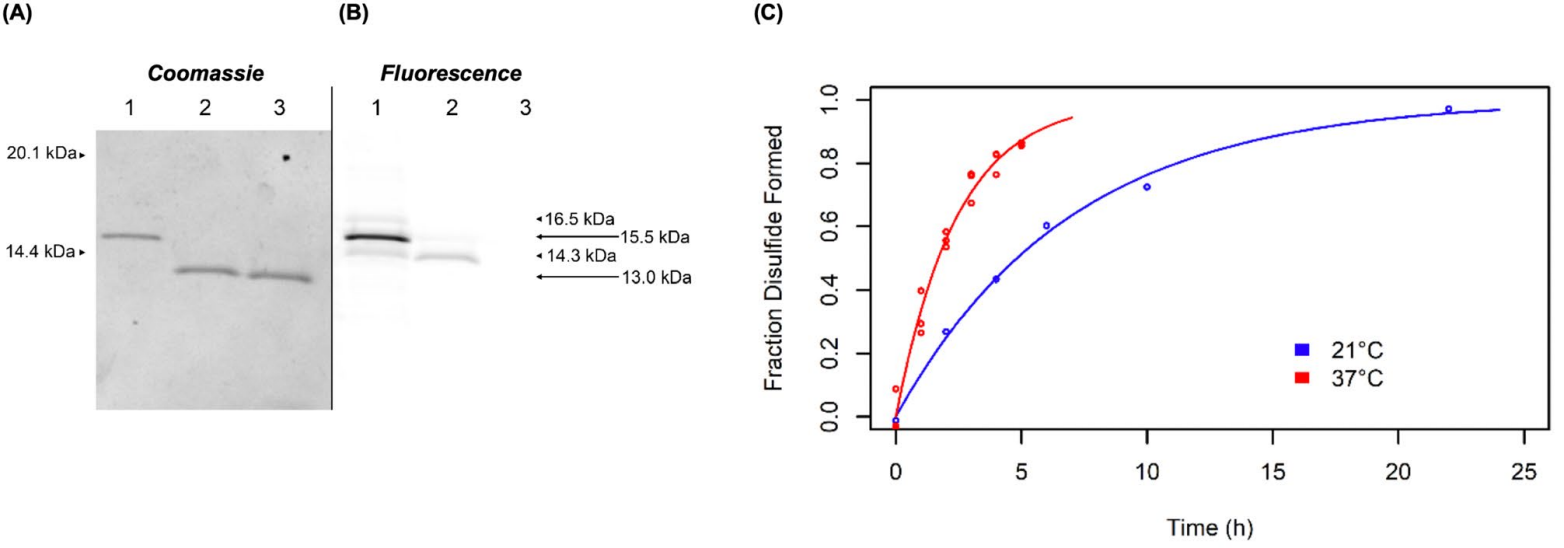
Formation of the disulfide bond in M88 and M112 studied by labelling with Alexa Fluor 633 maleimide. (**A**) Coomassie Blue-staining and (**B**) fluorescence-imaging of the same Tricine SDS-PAGE gel for M88. A reduced sample of M88 was used to start the experiment. Before initiating oxidation, an aliquot was taken and labelled with Alexa Fluor 633 maleimide as a positive control (lane 1, apparent mass = 15.5 kDa; theoretical mass = 15.7 kDa). Oxidized glutathione (10 mM) was added to the original, reduced sample to promote disulfide formation. After 2 min, Alexa Fluor 633 maleimide was added to another aliquot (lane 2). Lane 3 is unlabelled M88 (apparent mass = 13.0 kDa; theoretical mass 13.1 kDa). The very low amount of fluorescence measured for the band corresponding to the higher molecular mass in lane 2 indicates that approximately 0.1% of the protein contained reactive thiol groups that could react with the label, thus indicating efficient disulfide formation in less than 2 min. (**C**) M112 disulfide formation rate in the presence of 10 mM GSSG at 21 °C (blue) and 37 °C (red). Reduced M112 was treated with oxidized glutathione to promote disulfide formation. At the times indicated, an aliquot was taken and labelled with Alexa Fluor maleimide to assess free Cys by Tricine SDS-PAGE. Fluorescence intensity was then measured to quantify the extent of disulfide formation.

To monitor the conversion of the reduced form of M88 to the oxidized form with the formation of an intramolecular disulfide bond between Cys-49 and Cys-86, Alexa Fluor 633 maleimide was used as a probe for the presence of reactive thiol groups. In the reduced state, each of the two free thiol groups of M88 appears to react with a single molecule of Alexa Fluor 633 maleimide, generating a doubly-labeled protomer with an apparent increase in molecular mass of ~ 2.5 kDa that is easily detected using SDS-PAGE and close to the 2.6 kDa increase expected based on the mass of the fluorophore and linker added by the maleimide reagent (Fig. 2A lane 1). At lower contrast, an additional fluorescent band is visible at the position expected for the addition of one label (Fig S1), but the observation that this band is also present after reacting the AlexaFluor maleimide reagent with wild-type streptavidin (Fig S2), which contains no Cys, suggests that it is an unrelated side-reaction. For this reason, and the fact that this band is of relatively low fluorescent intensity and is undetectable by Coomassie staining, it was ignored in the analysis of disulfide bond formation.

With the labeling reagent at ~ 1,000-fold molar excess relative to the target thiol groups, this method was used to quantify the amount of free thiol groups and to indirectly infer the extent of disulfide bond formation. In the absence of exogenous oxidizing agents, reduced M88 showed no appreciable change in free Cys over 3 h (data not shown), indicating the relative inefficiency of disulfide bond formation with dissolved oxygen as the oxidizing agent. In contrast, the addition of 10 mM oxidized glutathione (GSSG) to 3 μM M88 promoted very efficient disulfide bond formation, with no free thiol groups detectable after 2 min of oxidation and labeling at 21 °C (Fig. 2A, lane 2; Supplementary Figs. S1 and S2). Because the labeling of M88 as an indirect indicator of disulfide bond formation is dependent on both the rate of disulfide bond formation and the rate of maleimide reacting with Cys, this experiment only establishes a lower limit for the rate of disulfide bond formation. The rapid and efficient formation of this disulfide bond is expected to be important for many practical applications.

### Oxidized M88 binds biotin-4-fluorescein (B4F) tighter than wild-type streptavidin

**Figure 3:**
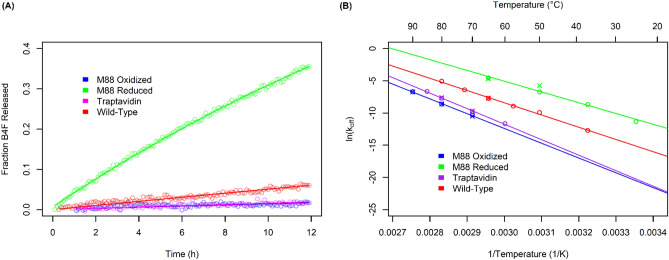
Dissociation of B4F from both reduced and oxidized M88, as well as from wild-type streptavidin and traptavidin at 25 °C. (**A**) B4F was added to an excess of streptavidin, binding to which causes fluorescence quenching. An excess of unlabelled biotin was added to prevent re-association of B4F and the increase in fluorescence was monitored over time. (**B**) Arrhenius plot showing how the rates of biotin dissociation from M88 (oxidized and reduced), traptavidin and wild-type streptavidin depend on temperature. The same competitive assay as in (**A**) was conducted but at a range of temperatures. Data is plotted as ln(k_off_) against 1/T, with the Celsius scale (top axis) added to assist with data interpretation. At temperatures where duplicate measurements were taken, both ○ and × symbols are used to assist with data visualization.

The rate constants for the association and dissociation of a fluorescent conjugate of biotin, biotin-4-fluorescein (B4F), were measured to estimate the binding affinity for biotin^13,30^. The change in quenching of B4F between the bound and unbound states allows for the measurement of progress curves indicating the rates of B4F binding and release over the range of seconds to minutes over a broad range of temperatures (21–90 °C). Starting with B4F bound to a large molar excess of streptavidin or its muteins, classic first-order dissociation kinetics and pseudo-first order association kinetics were observed between streptavidin and the B4F reporter. The extremely low rate of dissociation of B4F from the oxidized form of M88 at 25 °C (no change in B4F fluorescence measurable after 12 h) is similar to wild-type streptavidin and traptavidin (Fig. 3A), making it very challenging to accurately determine the rate of dissociation at room temperature. As a result, more accurate measurements of the rates of dissociation for M88, traptavidin and wild-type streptavidin were determined under comparable conditions over a range of temperatures extending between 25–90 °C. These measurements reveal linear and nearly parallel Arrhenius plots that indicate a consistent mechanism with roughly the same activation energy for dissociation over the range of temperatures evaluated. The oxidized form of M88 shows the lowest rate of dissociation that is approximately half the rate shown by traptavidin (Fig. 3B). Notably, kinetics could be measured at 90 °C only for oxidized M88, because traptavidin shows signs of irreversible denaturation soon after its addition at this temperature, whereas oxidized M88 appeared to be stable (data not shown). Within the error of the extrapolation procedure used to estimate the rate of, oxidized M88 and traptavidin appear to have approximately equal rates of dissociation at 25 °C that are 225 and 169 times lower than wild-type streptavidin, respectively (Table 2). These are two of the strongest non-covalent protein–ligand interactions known.

**Table 2:** Summary of binding kinetics between streptavidin variants and B4F at 25 °C.

	k_on_ (M^-1^ s^-1^)	k_off_ (s^-1^)	K_D_ (M)	t_1/2_
M88 oxidized	4.6 ± 0.3 × 10^5^	1.2 ± 0.4 × 10^–9^	2.6 ± 0.9 × 10^–15^	18.3 ± 6.1 years
M88 reduced	8.5 ± 0.5 × 10^6^	1.2 ± 0.06 × 10^–5^	1.4 ± 0.1 × 10^–12^	16.0 ± 0.8 h
M112 oxidized	1.7 ± 0.1 × 10^7^	4.1 ± 0.07 × 10^–4^	2.4 ± 0.1 × 10^–11^	28.2 ± 0.5 min
M112 reduced	4.4 ± 0.5 × 10^7^	6.5 ± 0.03 × 10^–6^	1.5 ± 0.2 × 10^–13^	1.23 ± 0.01 days
Traptavidin	2.3 ± 0.04 × 10^6^	1.6 ± 0.5 × 10^–9^	7.0 ± 2.1 × 10^–16^	13.7 ± 4.3 years
Wild-type	5.4 ± 0.5 × 10^7^	2.7 ± 0.5 × 10^–7^	5.0 ± 1.0 × 10^–15^	29.7 ± 5.5 days

Dissociation rate constants for M112 and reduced M88 were determined by nonlinear least-squares curve fitting of the experimental data (Figs. 3A, 6E). For oxidized M88, traptavidin and wild-type streptavidin, k_off_ was extrapolated from the Arrhenius plot (Fig. 3B). Measured rate constants are given ± standard error. Dissociation constant (K_D_) was calculated as k_off_/k_on_. Half-life (t_1/2_) was calculated as ln(2)/k_off_.

**Figure 4:**
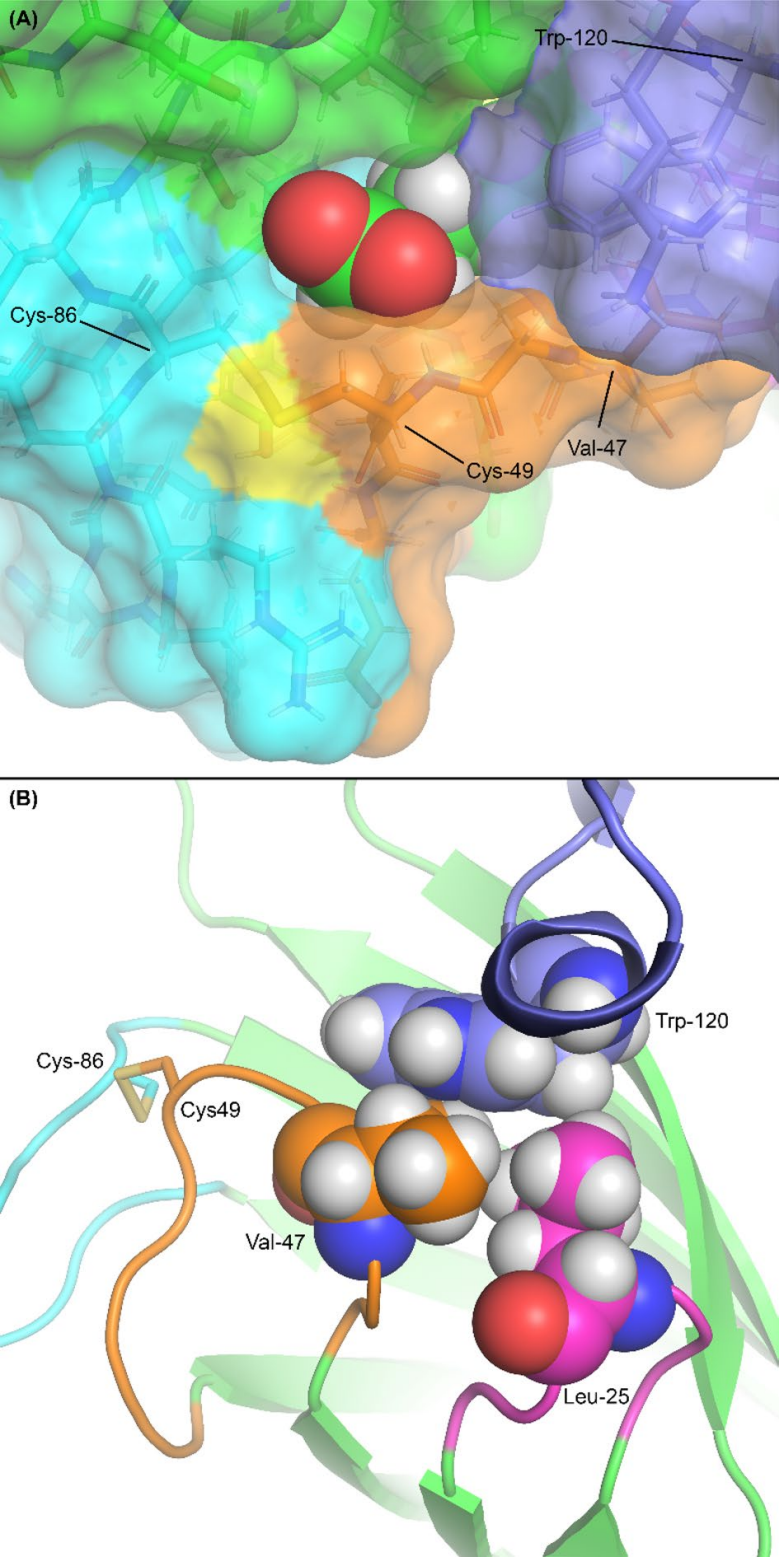
Overview of the M88 binding site. Molprobity was used to add hydrogens^49^. Loops are coloured the same as in Fig. 1 (loop 1–2 magenta, loop 3–4 orange, loop 5–6 cyan, loop 7–8 blue). The sulfur atoms of the two cysteine residues are coloured yellow. In this figure, the blue coloured loop 7–8 is that from the adjacent subunit in the tetramer which forms the complete binding pocket. (**A**) Surface representation looking down on the biotin binding site, with biotin shown as spheres. The critical steric barriers to biotin dissociation are loop 3–4 (orange), constrained in the closed conformation by the Cys-49/Cys-86 disulfide, and Trp-120. (**B**) Detail of the hydrophobic contacts between Val-47 of loop 3–4, Leu-25 of loop 1–2 and Trp-120 of loop 7–8 thought to be responsible for M88′s increased thermostability in the absence of biotin.

Comparison of the M88 structure with other high-resolution crystal structures of streptavidin suggests the molecular basis for this high-strength interaction. In wild-type apo-streptavidin, loop 3–4 is comparatively flexible and is generally either unobservable due to disorder or found in an open conformation (Fig. 1A). In biotin-bound structures, the loop is found in the closed conformation and sterically inhibits biotin dissociation. Related to this, traptavidin’s loop 3–4 has been shown to be in the closed conformation even without biotin bound, and its increased rigidity thus likely explains traptavidin’s higher affinity^15^. Previous studies using high-speed force spectroscopy and molecular dynamics simulations suggest that subtle movements of the apical portion of the 3–4 loop around the region of Gly-48 are necessary for biotin escape^31^. The proximity of M88′s Cys-49 to this portion of the loop means that disulfide formation would be expected to constrain movement around this critical region of loop 3–4 (Fig. 4A).

Although homologs of streptavidin with naturally occurring disulfide bonds in positions near the location of the disulfide bond in M88 have previously been studied, the effects of disulfide bond formation and reduction on biotin binding have not been thoroughly characterized. In the homologs rhizavidin, shwanavidin and hoefavidin, the dissociation constants for the weaker binding biotin analog 2-iminobiotin are only 1.5 to 2.5 times higher than for wild-type streptavidin, despite the lack of a residue equivalent to the Trp-120 residue shown to play an important role in biotin binding and release^26,28,32^. The disulfide bond in these proteins has been proposed to compensate for the loss of Trp-120 by reducing the conformational flexibility of loop 3–4. Although the binding kinetics of these homologs have not been studied under reducing conditions, double Cys to Ala mutations in shwanavidin and rhizavidin significantly increase the equilibrium dissociation constant. Our results indicate that the disulfide bond formed in M88 has a similar effect on its structure and function as the disulfide bonds found in somewhat distant homologs. In combination with the additional stabilizing effects of Trp-120, the addition of the engineered disulfide bond in M88 explains the substantially decreased rate of biotin dissociation when compared to wild-type streptavidin. The increased rigidity of loop 3–4 due to the formation of the disulfide bond is also reflected in the 117-fold and 18.5-fold decreases in the rate of biotin association for oxidized M88 when compared to wild-type streptavidin and the reduced form of M88, respectively (Table 2).

### M88 binding affinity is redox dependent

As expected, the disruption of the disulfide bond in M88 by treatment with mild reducing agents results in the weakening of biotin binding and a dramatic 10,000-fold increase in the rate of dissociation of B4F from reduced M88 at 25 °C (Table 2). Disruption of the disulfide bond likely increases the flexibility and dynamics of loop 3–4, allowing this loop to adopt a more open conformation required for biotin to dissociate from the binding pocket. This 10,000-fold increase in rate decreases the half-life of the biotin complex in reduced M88 to 16 h at 25 °C, which unfortunately is still too slow for many important practical applications, *e.g.*, isolation of biotinylated peptides and single-stranded nucleic acids. However, the steep dependence of the rate on temperature indicates that increasing the temperature to modest values is a simple and effective approach to increase dissociation rates for many practical applications. At 37 °C, for example, the half-life is reduced to 1.3 h and at 65 °C it is further reduced to 53 s (Fig. 3B). The high thermal stability of M88 indicates that increasing temperature to these or even higher levels would not lead to an appreciable amount of irreversible denaturation in the reduced form of M88.

Given that disulfide bond reduction in M88 increases the dissociation rate ~ 40-fold over wild-type streptavidin at 25 °C (Table 2), the conversion of Asn-49 and Ala-86 to Cys appears to reduce the binding affinity for biotin. In wild-type streptavidin, Asn-49 is a major contributor to direct binding interactions with biotin primarily due to hydrogen bonding between the valerate carboxylate group of biotin and the backbone amide group of Asn-49^6^. Furthermore, the side chain of Asn-49 forms two hydrogen bonds with Arg-84 of loop 5–6, likely stabilizing the closed conformation of loop 3–4 over the biotin binding pocket. In oxidized M88, the backbone hydrogen bond distance between Cys-49 and biotin appears to be very similar to that of Asn-49 in wild-type streptavidin. Additional analysis of the direct binding interactions between biotin and the oxidized form of M88 was performed using the Molegro Molecular Viewer^33^. This analysis indicates that there are no major changes in biotin binding for the oxidized form of M88 when compared to wild-type streptavidin. However, the side chain interactions are likely lost, or at least weakened, when M88 is in both the oxidized and reduced states, which likely have a destabilizing effect on the closed conformation of loop 3–4, thus explaining the decreased affinity of reduced M88 for biotin relative to wild-type streptavidin. This is supported by studies in which Asn-49 was replaced by non-natural amino acids, leading to a qualitative weakening of biotin binding affinity^34,35^. The replacement of Ala-86 with Cys has not been studied previously for streptavidin.

### M88 is even more thermostable than wild-type streptavidin

**Figure 5:**
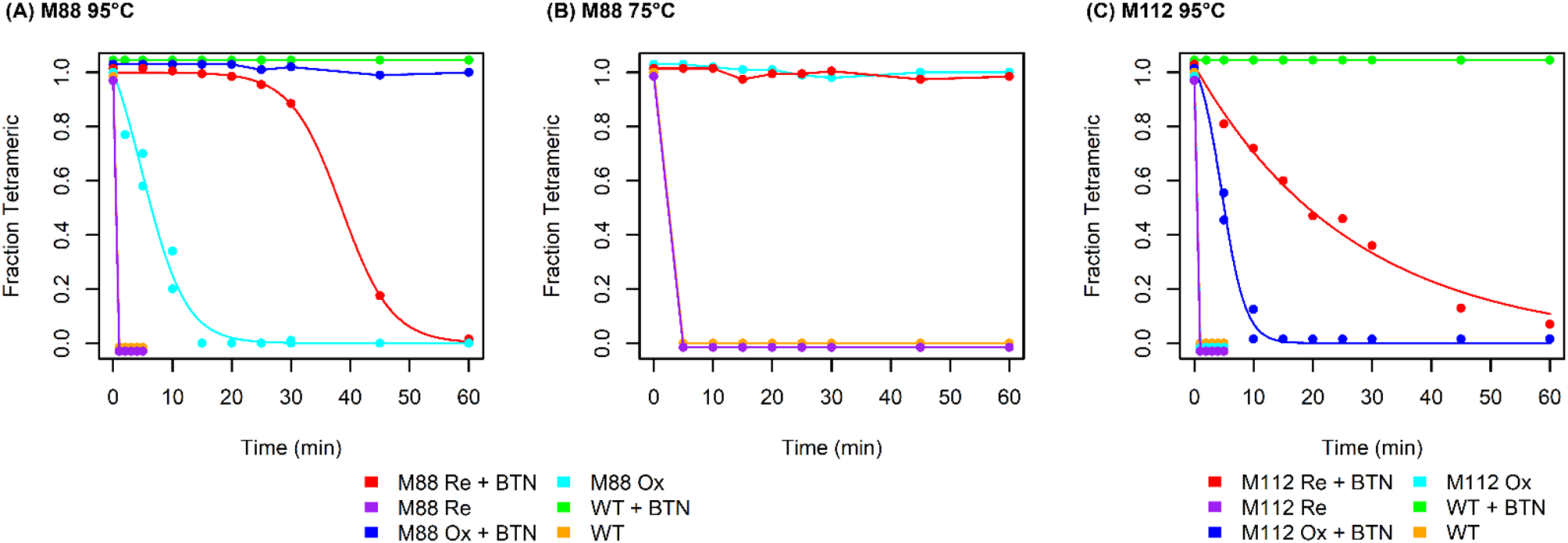
Thermostability of wild-type streptavidin, (**A**) M88 at 95 °C, (**B**) M88 at 75 °C and (**C**) M112 at 95 °C. Samples were heated in the presence of SDS with or without biotin. After treatment, folded tetramers were resolved from denatured monomers by SDS-PAGE. Band intensity was estimated with ImageJ software to determine the fraction of protein that did not denature. Each series is shifted slightly up or down for clarity. Wild-type data are the same in panels **A** and **C**. In the legends, Re and Ox refer to the reduced and oxidized forms of the disulfide bond, respectively, and WT refers to wild-type streptavidin.

Wild-type streptavidin shows exceptional thermostability, which is even further stabilized by biotin binding: the melting temperature increases from 75.5 °C in apo-streptavidin to 112.2 °C for the streptavidin–biotin complex^36^. Even in the presence of the strongly denaturing detergent sodium dodecyl sulfate (SDS), streptavidin remains tetrameric and bound to biotin when subjected to boiling temperatures^37^. In the presence of biotin at 95 °C, oxidized M88 behaves similarly to wild-type streptavidin in that no appreciable denaturation of the tetramer occurs over the course of a 60 min incubation (Fig. 5A). As expected, reduction of the disulfide bond does destabilize M88 and half of the sample appears to be irreversibly denatured by ~ 38 min even in the presence of biotin. When biotin is absent, both reduced M88 and wild-type streptavidin appear to be completely denatured in less than 1 min. Very interestingly, the M88 disulfide bond substantially stabilizes the protein in the absence of biotin, requiring ~ 6 min to denature half of the protein. This effect is even more pronounced for heating at 75 °C, where oxidized M88 without biotin remains tetrameric even after a 60 min incubation, whereas both the reduced form of M88 and wild-type streptavidin appear to denature completely within 5 min in the absence of biotin (Fig. 5B).

The extreme thermostability of streptavidin appears to depend in large part on Trp-120 of loop 7–8, as well as the contacts it forms with loop 3–4 and the biotin molecule primarily bound to the adjacent subunit in the tetramer^38^. M88′s thermostability in the absence of biotin suggests that the disulfide bond promotes the closure of loop 3–4 regardless of the presence of biotin, which promotes interactions between this loop and Trp-120, helping to contribute a substantial amount to the strength of intersubunit interactions. Specifically, Val-47 in the closed conformation of loop 3–4 appears to be in particularly close contact with Trp-120 of loop 7–8 and Leu-25 of loop 1–2 (Fig. 4B), forming a network of hydrophobic interactions that likely provides stabilization to the tetramer. While biotin promotes loop closure in wild-type streptavidin, this effect partially occurs in oxidized M88 even in the absence of biotin, since the disulfide bond appears to lock the loop in the closed position.

### The disulfide bond in M112 induces structural perturbations that lead to slow kinetics of disulfide-bond formation, weaker affinity for biotin and lower thermostability

**Figure 6:**
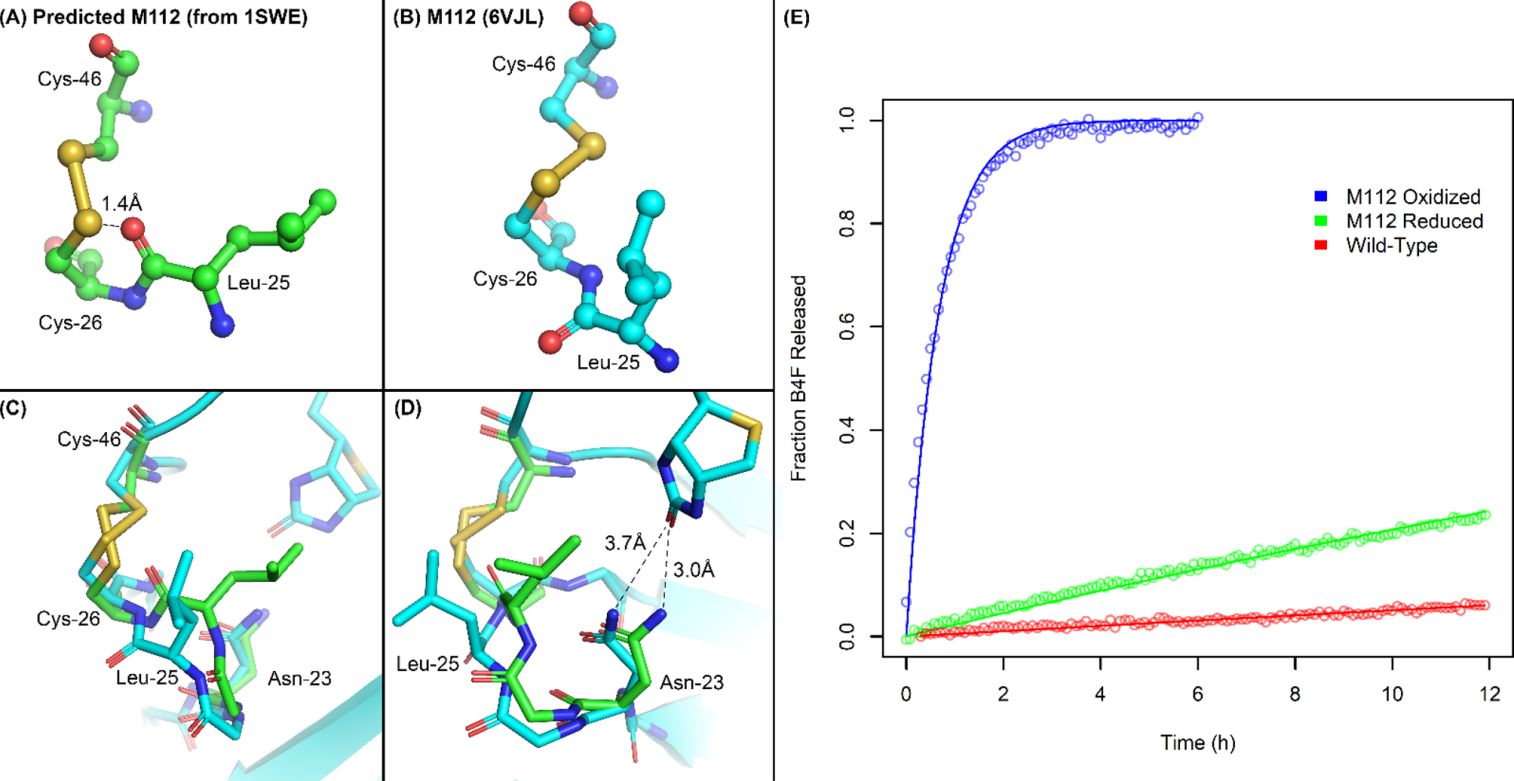
Structural and kinetic characterization of M112. (**A**–**D**) Comparison of the M112 disulfide and loop 1–2 with that of wild-type streptavidin (1SWE) after structural superposition. (**A**) Wild-type streptavidin with Cys mutations predicted by Disulfide by Design 2.0. Also shown is the distance between the sulfur of Cys-26 and the oxygen of the Leu-25 backbone carbonyl group, indicating a steric clash. (**B**) M112 disulfide and Leu-25. (**C**) The disulfide bonds in their structural context. (**D**) Same image as C rotated 60° about the y-axis. Shown are the interatomic distances between Asn-23 of both streptavidin and M112 with the biotin ureido oxygen. (**E**) Dissociation of B4F from both reduced and oxidized M112, as well as from wild-type streptavidin. B4F was added to an excess of streptavidin, binding to which causes fluorescence quenching. An excess of unlabelled biotin was added to prevent re-association of B4F and the increase in fluorescence was monitored over time. Wild-type data are the same as in Fig. 4A.

In contrast the stabilizing effects on the closed conformation of loop 3–4 seen in M88, the disulfide bond in M112 had an opposite and generally undesirable set of effects that destabilized loop 1–2. The crystal structure of oxidized M112 in complex with biotin shows that the detailed geometry of the M112 disulfide bond differs from the prediction provided by Disulfide by Design 2.0 arising from a steric clash between the sulfur of Cys-26 and the oxygen of the backbone carbonyl group of Leu-25 (Fig. 6A). The crystal structure reveals how a small but significant rotation along the Cys-26 N-C_α_ bond between Leu-25 and Cys-26 is able to resolve this clash (Fig. 6B). However, this change also leads to a conformational change in loop 1–2 (residues 23–27) that shifts the side chains of Leu-25 and Asn-23 away from the biotin binding site (Fig. 6C,D). The electron density corresponding to the side chain of Leu-25 is very weak and suggests an increase in disorder or conformational flexibility for the side chain in addition to a shift in its position away from the well-ordered contacts formed in the biotin-bound state of wild-type streptavidin (Supplementary Movie 1).

Consistent with the structural observations of strain induced by the formation of the disulfide bond in M112 and quite unlike the formation of the disulfide bond in M88, the M112 disulfide bond forms very slowly even in the presence of 1,000-fold molar excess of oxidized glutathione, with pseudo-first order rate constants of 0.14 ± 0.02 h^−1^ (21 °C) and 0.41 ± 0.06 h^−1^ (37 °C) (half-lives of 5 and 1.6 h, respectively) (Fig. 2B). The much slower rate of disulfide bond formation in M112 compared to M88 is likely related to the strain induced by disulfide bond formation and the destabilizing conformational change required for loop 1–2 to accommodate the formation of the new disulfide bond. Since Leu-25 and Asn-23 of this loop interact with biotin in wild-type streptavidin and presumably also in the reduced form of M112, the disruption of these interactions required by the formation of the disulfide bond in M112 help to account for some of the kinetic and thermodynamic barriers to disulfide formation.

The formation of the disulfide bond in M112 also increases the rate of dissociation for B4F ~ 60-fold and ~ 1,500-fold when compared to the reduced form of M112 and wild-type streptavidin, respectively (Fig. 6E) (Table 2). Comparing the structures of wild-type streptavidin with the oxidized form of M112 suggests that the rigidity of loop 1–2 and the formation of close contacts between biotin and the loop, especially Leu-25 and Asn-23, make important contributions for binding affinity. While Leu-25 makes significant van der Waals contacts with biotin in wild-type streptavidin, these interactions are lost in M88 as loop 1–2 moves away from the binding site. This movement of loop 1–2 and Leu-25 away from biotin also allows greater solvent accessibility to biotin, with the electron density suggesting the presence of partially ordered water molecules present in the region occupied by Leu-25 in the wild-type streptavidin–biotin complex. Additionally, the hydrogen bond between the nearby residue Asn-23 and the biotin ureido oxygen is lengthened to 3.7 Å compared to 3.0 Å in wild-type streptavidin (Fig. 6D); the disruption of this hydrogen bond has been predicted to be an early step in biotin dissociation from molecular dynamics simulations^39^. Gly-26, Ala-46 and the equivalent Cys residues in M112 are not predicted to make a significant contribution to direct binding interactions with biotin, but indirect effects from these changes likely contribute towards the 21-fold increase in the rate of biotin dissociation from reduced M112 compared to wild-type streptavidin (Table 2).

The thermostability of M112 is also consistent with the observed structural changes and biotin-binding kinetics, with both oxidized and reduced M112 being less thermostable than both wild-type streptavidin and M88. When heated to 95 °C in the presence of SDS, both the reduced and oxidized forms of M112 in the absence of biotin denatured within the first five minutes, similar to wild-type streptavidin and the reduced form of M88. In stark contrast with M88, however, the addition of biotin only slightly increased the stability of M112, and both oxidized and reduced M112 were much less stable than wild-type streptavidin in the presence of biotin (Fig. 5C). This is consistent with the observed decreased stability of the M112-biotin complex as well as with the roles of Leu-25, Val-47, and Trp-120 deduced from the M88 crystal structure and thermostability data. Perhaps the single most important structural perturbation leading to decreased thermostability in M112 is the increased flexibility of loop 1–2 arising from the shift in position of Leu-25 away from the interaction partners seen in wild-type streptavidin and M88.

When compared with the effects seen in M88, these results show how the formation of a disulfide bond can induce effects opposite to those desired, namely destabilizing the binding of biotin and increasing the rate of dissociation. As a result, M112 still displays redox-switchable affinity for biotin, but the difference between oxidized and reduced forms is much lower (~ 60-fold) than for M88, which shows ~ 10,000 fold difference at 25 °C. In addition, the slow rate of disulfide bond formation in M112 would also make it much less useful for most practical applications.

### Advantages and disadvantages of M112 and M88 for practical applications compared with wild-type streptavidin and previously characterized mutants

After characterizing the effects of introducing two disulfide bonds individually into the N- and C-terminal sides of loop 3–4 in streptavidin, we discovered a number of strikingly opposite effects on biotin binding affinity when comparing the behavior of the two mutants M88 and M112. The series of structural perturbations necessary to accommodate the disulfide bond introduced into the N-terminal side of loop 3–4 in M112 results in an undesirable increase in the rate of dissociation for biotin and an undesirable decrease in thermostability compared to reduced M112 and wild-type streptavidin. In addition, the slow rate of disulfide bond formation in this mutant reduces its utility for many practical applications that require a rapid switch between reduced and oxidized states.

In contrast, the introduction of a disulfide bond into the C-terminal side of loop 3–4 leads to several novel and desirable effects that make M88 uniquely useful as a switchable biotin-specific binding protein. M88 has very high binding affinity and low rate of biotin dissociation in the oxidized state, but when the disulfide bond is disrupted in the reduced state, there is a dramatic 19,000-fold increase in the rate of dissociation (at 21 °C). Moreover, the rate of dissociation in reduced M88 is ~ 70-fold faster than for wild-type streptavidin. Finally, the rates of disulfide bond formation and breakage under mild conditions of oxidation and reduction also appear to be quite fast.

Another major advantage of M88 over wild-type streptavidin is that the formation of the disulfide bond in the oxidized, disulfide-bonded state decreases the rate of biotin release 225-fold compared to wild-type streptavidin. This rate of dissociation is one of the lowest measured for non-covalent interactions and is slightly lower than for traptavidin, an engineered variant of streptavidin containing stabilizing non-covalent interactions at the base of loop 3–4^13^ (Table 2). Because traptavidin has a fivefold higher rate of association for biotin compared to oxidized M88, it still has a lower equilibrium dissociation constant. In addition, the insensitivity of traptavidin to redox conditions make it more useful than M88 for maintaining tight binding to biotin under reducing conditions. The greatest advantage of M88 over previously available forms of streptavidin, however, lies in its unique combination of extremely tight binding under oxidizing conditions, a 19,000-fold increase in the rate of dissociation from biotin under reducing conditions and the fast, mild and economical process required to reversibly switch between these two states using thiol-specific reducing agents. Although our group previously reported the use of intermolecular disulfide bonds to allow for the redox-switchable binding of an engineered form of streptavidin to streptavidin-binding peptides^20^, M88 has the advantage that it is compatible with any biotinylated molecule, making it a much more general, flexible and economical solution for most applications.

Although the rate of dissociation for biotin is ~ 40-fold faster from the reduced form of M88 compared to wild-type streptavidin at room temperature, the 24-h half-life is still too low for many practical applications. However, a modest increase in temperatures to 45 °C, for example, decreases the half-life for dissociation to 20 min. The higher rates of dissociation at slightly elevated temperatures would allow M88 to provide uniquely useful binding properties for many applications, including the regeneration of biosensors and the release of captured biotinylated peptides, glycans and nucleic acids, where both extremely tight binding and the efficient release of biotinylated ligands are required.

For applications requiring faster rates of dissociation at room temperature, M88 is a prime candidate for further engineering. An extensive body of research on streptavidin has already identified a broad selection of mutations that increase the rates of dissociation to different extents that can be tailored to the needs of specific applications. If the ~ 19,000-fold difference in dissociation rates of the reduced and oxidized forms of M88 at room temperature is preserved, mutants based on M88 may allow even faster dissociation in the reduced form while still retaining high binding affinity in the oxidized state. Our preliminary work along these lines suggests that second-generation mutants based on M88 can likely be created with the ability to release biotinylated ligands over the course of a few minutes at room temperature or slightly higher temperatures. These advances will further expand the utility of the streptavidin–biotin system for an even wider range of applications where an extremely tight binding state and rapid release can be reversibly controlled by mild oxidation and reduction reactions.

A possible limitation of the use of disulfide-gated switches to regulate binding affinity is that mild reducing agents need to be added to induce ligand dissociation. This limitation may be problematic for applications such as the elution of proteins or protein complexes which contain native disulfide bonds that could be irreversibly disrupted by the addition of reducing agents. Although this could be an important limitation of the use of a disulfide-gated switch, it is also likely that many native disulfide bonds could likely be reformed efficiently once the reducing agents have been removed. Optimizing the conditions required for the reduction of the disulfide bond in M88 would likely be beneficial in such cases to maximize the release of biotinylated ligands while minimizing the disruption of native disulfide bonds.

## Materials and methods

### Construction of expression vectors

Synthetic genes for SAVM88CV, SAVM112CV and SAVM112 were produced and cloned into the pUCSP plasmid by Bio Basic Inc. Canada. Each plasmid was individually digested with *Nde*I/*Blp*I (New England BioLabs) to release the insert which was subsequently cloned into *Nde*I/*Blp*I digested pET29B plasmid (Novagen).

A synthetic gene for SAVM88 was produced and cloned into the pUC57 plasmid by Bio Basic Inc. Canada, generating pUC57-FLSAVM88. This plasmid was digested with *Hin*dIII (Thermo Fisher Scientific) and *Eco*RV (New England BioLabs) to release a 503-bp fragment carrying the full-length SAVM88 gene. This fragment was inserted to pUB18-SAV(SN)^40^ digested with *Hin*dIII and *Eco*RV cut to generate pUB18-FLSAVM88.

### Protein expression and purification

*Bacillus subtilis* WB800^40,41^ was used for secretory expression of full-length M88. A log-phase plate culture was used to inoculate 100 mL super rich medium^42^ with 10 μg/mL kanamycin. Cells were cultured at 30 °C for 16 h and then pelleted by centrifugation at 8,000 *g*. Phenylmethylsulfonyl fluoride (PMSF) was added to 0.1 mM. To minimize the irreversible oxidation of Cys, ethylenediaminetetraacetic acid (EDTA) and β-mercaptoethanol were added at 1 and 10 mM, respectively. Finally, ammonium bicarbonate was added at 50 mM and the pH titrated to 11 with sodium hydroxide. The culture supernatant was then applied to a 1 mL bed volume 2-iminobiotin agarose column by peristaltic pump at a flow rate of 1 mL/min. The column was washed with 15 mL 50 mM ammonium bicarbonate, 500 mM sodium chloride, 1 mM EDTA, 10 mM β-mercaptoethanol, pH 11 and the protein was eluted with 50 mM sodium acetate, 500 mM sodium chloride, 1 mM EDTA, pH 4.

The core-form streptavidin muteins, with the N- and C-terminal tails trimmed (comprised of residues 14–136 from the mature form of wild-type of streptavidin), as well as full-length M112, were expressed as inclusion bodies in *Escherichia coli* BL21(DE3). Three millilitres of an overnight broth culture were added to 150 mL LB with 30 μg/mL kanamycin, grown at 37 °C for 3 h before being induced with 0.4 mM isopropyl β-D-1-thiogalactopyranoside (IPTG) and cultured for an additional 5 h. Pelleted cells from three 150 mL cultures were resuspended with 6 mL Tris-buffered saline (TBS; 20 mM Tris–HCl, 50 mM sodium chloride, pH 8) including 1 mM EDTA and then incubated with 0.1 mg/mL lysozyme for 30 min at 37 °C. Cells were lysed by sonication, and the inclusion bodies were pelleted. Streptavidin muteins were refolded by the method of rapid dilution as previously described^43^. Briefly, inclusion bodies were resuspended and washed three times with 20 mL 50 mM sodium acetate, 1.5 M sodium chloride, 1 mM EDTA, 1% Triton X-100, pH 4 and then three times with the same buffer lacking Triton and with 50 mM sodium chloride. The final pellet was resuspended in 4 mL 6 M guanidinium chloride pH 1.5 + 2.5 mM tris(2-carboxyethyl)phosphine (TCEP) and then centrifuged to remove any remaining insoluble debris. The supernatant was then added dropwise to 250 mL 50 mM dibasic sodium phosphate, 50 mM ammonium bicarbonate, 100 mM sodium chloride, 1 mM EDTA, 10 mM β-mercaptoethanol, pH 7.4 at 4 °C with fast stirring. This mixture was then centrifuged, and the supernatant was titrated to pH 11 with sodium hydroxide and purified on 2-iminobiotin agarose as described above.

For all proteins, anion exchange chromatography was performed as a final polishing step. Elution fractions from 2-iminobiotin agarose were combined, concentrated and exchanged into 20 mM Tris, 1 mM EDTA, 5% glycerol, pH 8 (QA buffer) by centrifugal filtration and applied to a 1 mL HiTrap Q HP column using an ÄKTA purifier. Streptavidin was eluted using a gradient of 0 to 30% QA to QB buffer (QA + 1 M sodium chloride) and fractions from the sharp initial peak were pooled.

Protein concentration was determined by measuring absorbance at 280 nm with a NanoDrop spectrophotometer using extinction coefficients estimated by ExPASy ProtParam. Because full-length M88 was expressed as a soluble protein in the presence of biotin in the culture medium, the concentration of free binding sites was determined by cumulative titration with B4F as previously described^30^. Wild-type streptavidin was obtained as a lyophilized powder from Bio Basic. Traptavidin was obtained from Kerafast.

### Crystallization and structure determination by X-ray crystallography

Oxidized, biotin-bound, core-form M88 was crystallized by the hanging drop vapour diffusion method using 1.5 μL of 8% glycerol, 21% PEG 3,350, 100 mM Bis–Tris pH 7.5 combined with 1.5 μL 5.6 mg/mL core M88. Oxidized, biotin-bound, full-length M112 was similarly crystallized by mixing 1.5 μL of 28% PEG 4,000, 0.15 M ammonium sulfate, 50 mM Bis–Tris pH 7.5 with 1.5 μL of 9.5 mg/mL M112. Single crystals were flash-cooled in liquid nitrogen and shipped to Beamline 12–2 at the Stanford Synchrotron Radiation Lightsource (SSRL) and Beamline 08B1-1 at the Canadian Macromolecular Crystallography Facility at the Canadian Light Source to screen crystals for the quality of diffraction prior to data collection. The best data sets were measured from crystals sent to SSRL. For the oxidized complex of M88 bound to biotin, diffraction images were indexed and integrated using MOSFLM^44^. Scaling and space group determination were performed using SCALA and POINTLESS from the CCP4 suite^44^. For the oxidized complex of M112 bound to biotin, diffraction images were indexed and integrated using XDS^45^. Scaling was performed using XSCALE and space group determination was performed using POINTLESS from the CCP4 suite^44^. Scaled intensity measurements from both crystals were converted to structure factor amplitudes using TRUNCATE. For the M88 complex, initial phases were calculated using the molecular replacement procedure implemented in PHASER^46^, starting with the coordinates of 1SWE (chains A and B) as the search model. Six copies of the dimer search model were positioned by the automated rotation and translation search procedure, generating four canonical tetramers. For the M112 complex, PHASER was used to position the coordinates of 1SWE (chain A) as the search model. A single copy of the monomer search model was positioned, with the canonical tetramer being generated by a crystallographic symmetry four-fold rotation operator. REFMAC^47^ was used for positional and temperature factor refinement, and COOT^48^ was used for the inspection of electron density maps and manual model building. Molprobity was used to evaluate and correct modeling errors^49^.

### Rate of disulfide bond formation

Protein disulfide bond formation was catalyzed by thiol-disulfide exchange with either oxidized glutathione (GSSG) or 2,2′-dipyridyldisulfide. Disulfide bonds were inferred by selectively labelling free Cys with Alexa Fluor 633 C5 maleimide (Invitrogen) and subsequent separation by Tricine SDS-PAGE^50^. Detection is based on both fluorescence and a mobility shift due to the large size of these labels (~ 1.3 kDa each, ~ 2.6 kDa if both cysteines in M88 or M112 react). Ratios of reactants were based on those previously described for saturation labelling^51^. Because the N- and C-terminal tails are susceptible to degradation by trace proteases, leading to band heterogeneity in the gel, core-form versions of M88 and M112 were primarily used for this assay. In a total volume of 10 μL (buffered to pH 7.5 with 100 mM Tris–HCl), 6 pmol M88 or M112 (48 pmol thiol) was labelled with 4,800 pmol Alexa Fluor 633 maleimide for 45 min in the dark. As a positive control, an additional 6 pmol aliquot of protein was first reduced by incubation with 1,200 pmol TCEP for 45 min followed by Alexa Fluor labelling as described. β-mercaptoethanol was added to stop the reaction. To remove excess label, 3 μg bovine serum albumin was added as a carrier and the samples were precipitated with 95% ethanol followed by centrifugation. The dried pellet was resuspended in 12 μL formamide and 4 μL SDS-PAGE loading buffer, heated to 95 °C for 10 min and then run on a 16%T/5%C Tricine-SDS-PAGE gel. Immediately after the run, fluorescence was measured with excitation by 635 nm laser and emission collected through a 665 nm long-pass filter on a Typhoon FLA 9,500 laser scanner (GE Healthcare).

To measure the time course of disulfide formation, streptavidin muteins were reduced with TCEP which was subsequently removed by centrifugal filtration. The reduced protein was then mixed at a final concentration of 3 μM with 0.1 mM biotin and 10 mM GSSG in TBS. At each timepoint, a 4 μL aliquot of the reaction mixture was quenched/labelled with the addition of Alexa Fluor 633 C5 maleimide. The samples were prepared for electrophoresis and imaged as described above. Fluorescence intensity of each band was quantified using ImageQuant software and the amount off free Cys at each timepoint was determined relative to the initial amount. Given the large excess of oxidized glutathione, pseudo-first order kinetics were assumed and the data were fit to the single exponential ${-e}^{-kt}$.

### Biotin-4-fluorescein off-rate assay

Dissociation kinetics were measured with a competitive assay exploiting the fluorescence quenching of B4F upon binding to streptavidin^13,30^. In a total volume of 200 μL, 50 nM streptavidin, 10 nM B4F (Invitrogen), 50 mM NaCl, 1 mM EDTA, 20 mM Tris pH 8, were incubated for 30 min. Biotin was added to 0.1 mM to prevent B4F re-association and fluorescence was monitored using a Tecan Infinite M200 plate reader with 485 nm excitation/525 nm emission for 12 h at 25 °C. The maximum, with all B4F unbound, and minimum, with all B4F bound, fluorescence was determined with similar mixtures in the absence of either streptavidin or biotin, respectively, and the fraction of B4F released at each time point was calculated as $\%B4F Released=\frac{response-min}{max-min}\times 100\%$. For all proteins measured, release of B4F was biphasic with a rapid initial release phase followed by a more significant slow phase. The rapid phase was ignored and off-rate constants were determined by nonlinear least squares fitting of the data to the single exponential function $y=1- {e}^{kt}$. To confirm that re-association or secondary reactions between free biotin and B4F are not affecting the rates of fluorescence changes, dissociation rates were measured after adding biotin at a range of concentrations between 0.05–1.0 mM (Supplementary Fig. S3). The lack of variation in measured dissociation rates over the range of free biotin concentrations between 0.1–1.0 mM indicates that 0.1 mM biotin was sufficient to prevent re-association and did not induce secondary reactions affecting the change in quenching seen for B4F dissociation.

High-temperature kinetics were measured by the same competitive assay but instead using a Horiba Jobin–Yvon FluoroMax-4 spectrofluorometer. To limit evaporation and condensation within the cuvette, it was filled to the top with 4 mL of solution using the same concentrations of reagents as above. Fluorescence was initially measured without streptavidin, giving the maximum, unquenched signal. Streptavidin was then added and allowed to equilibrate until a constant fluorescent signal was achieved, and unlabelled biotin was added to start the experiment. At these higher temperatures, the rapid release phase was more apparent and accounted for by fitting the data to the double exponential function $y=max- {ae}^{{-k}_{a}t}-{be}^{{-k}_{b}t}.$ The linear Arrhenius plot was generated by plotting *ln(k*_*off*_*)* against *1/temperature*. A linear least squares fit to the function $ln\left({k}_{off}\right)=ln\left(A\right)- \frac{{E}_{a}}{RT}$ was used to extrapolate the dissociation constant at lower temperatures.

### Biotin-4-fluorescein on-rate assay

Association kinetics were also monitored by utilizing B4F. In 2 mL total volume, 0.1 nM B4F, 50 mM NaCl, 1 mM EDTA, 20 mM Tris pH 8, were equilibrated in the cuvette for 20 min at 25 °C. 20 μL streptavidin was added and measurements were started immediately on a Horiba FluoroMax-4 spectrofluorometer with 490 nm excitation/525 nm emission and 2 and 15 nm slit widths, respectively, for the monochromator. Protein concentrations used were: 0.4, 0.8, 1.6, 2.4, 3.2 nM for wt; 0.8, 1.6, 3.2, 6.4 nM for oxidized M112; 0.4, 0.8, 1.6, 3.2 nM for reduced M112; 0.77, 1.5, 3.1, 6.1 nM for reduced M88 and 6.25, 12.5, 25, 50 nM for traptavidin. For oxidized M88, measurements were done in 60 μL total volume using 10 nM B4F and 50, 100, 200 and 400 nM M88. On-rate constants were determined by nonlinear least squares fitting of the data to the integrated first order rate equation, under the assumption that binding was pseudo-first order.

### Thermostability assay

To assess thermostability, SDS was added to 2% to wild-type or previously oxidized M88 or M112 and was incubated in the presence or absence of 5 mM biotin and in the presence and absence of 200 mM β-mercaptoethanol for 15 min. The samples were heated at 75 or 95 °C for 1 h and aliquots taken every 5 min for the first 30 min, and then at 45 and 60 min. Samples from each time point were run on a 12% SDS-PAGE gel, which were than stained with Coomassie blue. Band intensity was quantified using ImageJ software and the fraction of protein that remained tetrameric was determined relative to time zero. The band intensities for a series of standards was determined to indicate the linear range for quantification.

### Software used for figure preparation

PyMOL (Schrödinger) was used to prepare the figures of molecular structures. R (https://www.R-project.org) was used for least-squares curve-fitting and graphical data analysis.

### Accession numbers

The coordinates and structure factors for the oxidized forms of M88 (6VJK) and M112 (6VJL) have been deposited in the Research Collaboratory for Structural Bioinformatics (RCSB) Protein Data Bank (PDB).

## Supplementary information

Supplementary file1 (DOCX 3909 kb)

Supplementary file2 (MP4 7104 kb)

## Abbreviations

B4F: Biotin-4-fluorescein
EDTA: Ethylenediaminetetraacetic acid
GSSG: Oxidized glutathione
SDS: Sodium dodecyl sulfate
TCEP: Tris(2-carboxyethyl)phosphine
