# Supplementary material for: Engineering a disulfide-gated switch in streptavidin enables reversible binding without sacrificing binding affinity
Source: Sci Rep. 2020 Jul 27;10:12483. doi: 10.1038/s41598-020-69357-5 (PMC7385176; doi:10.1038/s41598-020-69357-5)
Supplement: Supplementary file 1 — Supplementary file1 (DOCX 3909 kb) [file 41598_2020_69357_MOESM1_ESM.docx]

**Engineering a disulfide-gated switch in streptavidin enables reversible binding without sacrificing binding affinity**

Jesse M. Marangoni^a^, Sau-Ching Wu^a^, Dawson Fogen^a,b^, Sui-Lam Wong^a^, Kenneth K.S. Ng^a,c^

^a^Department of Biological Sciences, University of Calgary, Calgary, Alberta, Canada T2N 1N4

^b^Present address: Department of Molecular Biosciences and Bioengineering, University of Hawaii, Honolulu, HI, USA 96822

^c^Corresponding author; ngk@ucalgary.ca

**Supplementary Information (Movie legend and figures)**

**Supplementary Movie File 1 (Marangoni_Movie_Supplement1.mp4)**

Three views are shown for the structure of the oxidized form of M112 around the region of Leu-25 and the newly introduced disulfide bond formed between Cys-26 and Cys-46. The difference electron density map (green lines) is contoured at 3.2 standard deviations above the mean value of the electron density of the map using ||***F_o_***|-|***F_c_***|| amplitudes and phases calculated from a model of M112 in which residues 24-26 and 46 were omitted prior to 20 rounds of refinement and electron density map calculation. The movie was prepared using COOT [46].


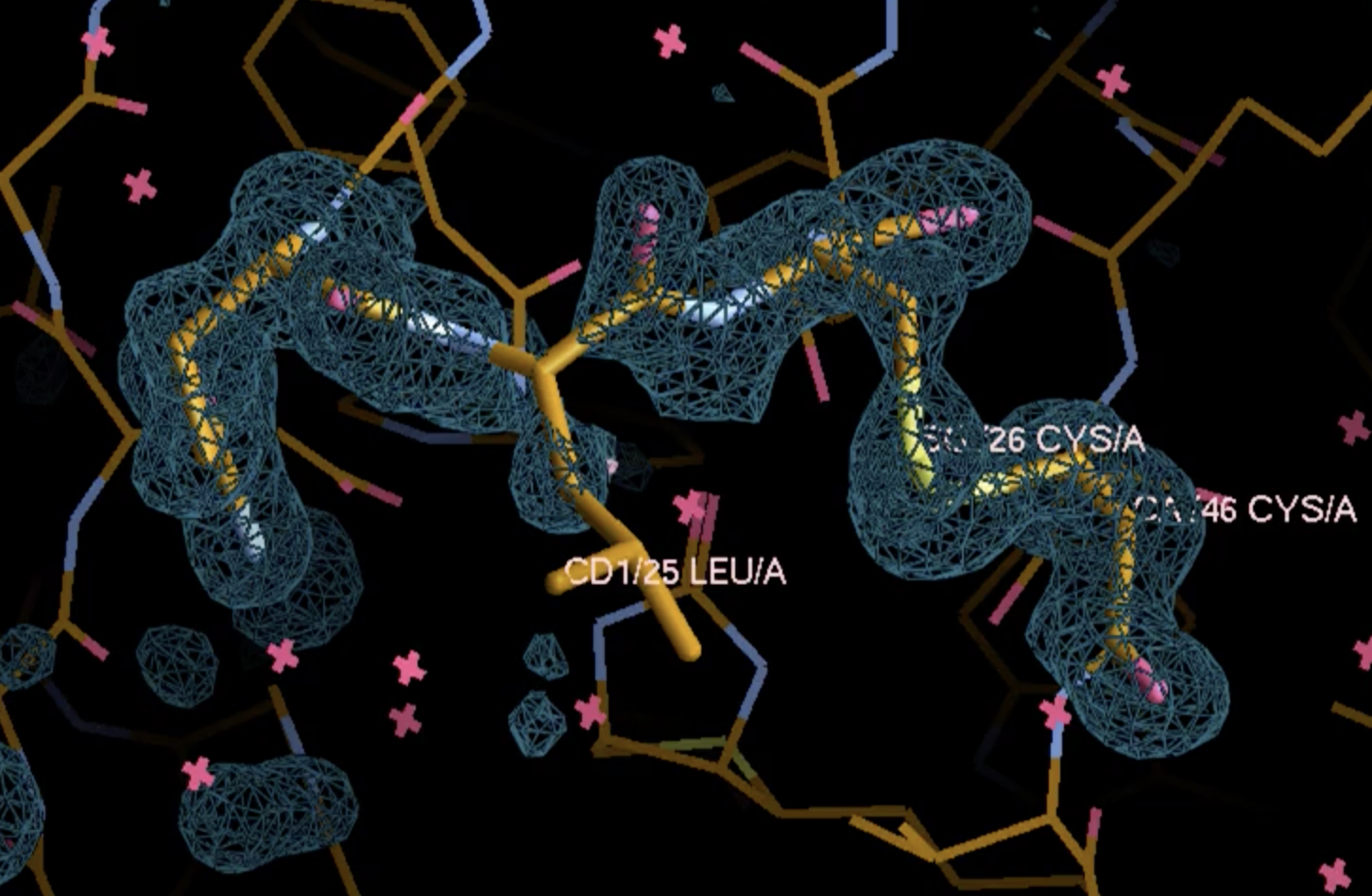


**
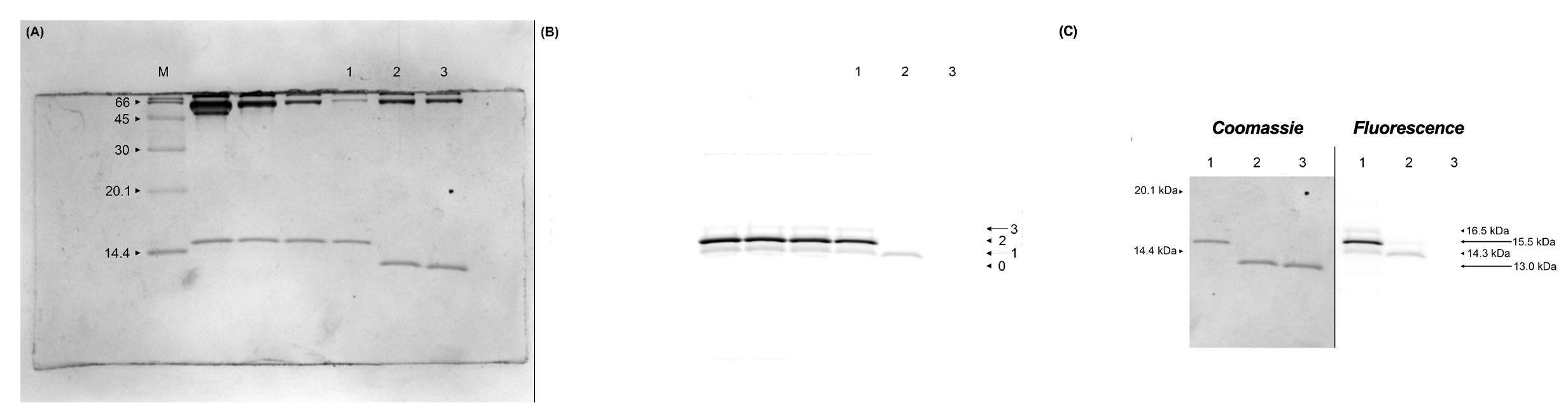
**

**Supplementary Figure S1: Uncropped versions of the Coomassie (A) and fluorescent (B) gel images that were applied to generate panels A and B of Fig 2.** The resulting panels A and B of Fig 2 is shown here as panel C in Figure S1. Lanes in panel A of Figure S1 are numbered as in Fig 2A, with the molecular weight marker labelled M and the molecular mass (kDa) of each band indicated to the left. Unlabelled lanes correspond to reactions from an unrelated experiment. The high molecular weight bands around 66 kDa and higher likely correspond to BSA added as a carrier during ethanol precipitation. The fluorescent image (panel B of Figure S1) is an uncropped version of that shown in Fig 2B. In addition to the major fluorescent band indicating two Cys labelled per M88 protomer (band 2 in lane 1), there are also minor fluorescent bands at positions consistent with 1 and 3 residues reacting with the Alexa Fluor maleimide label (bands 1 and 3 in lanes 1 and 2). Since a single M88 protomer has only 2 cysteine residues, the presence of band 3 suggests that another residue also reacts with the maleimide label. This is consistent with the observation that less reactive groups (likely amino groups) in wild-type streptavidin, which contains no Cys, also react with the maleimide label (Fig S2). Note that the relative intensities of the weak bands are misleadingly prominent in the lower contrast image, because the intensity of the major band is oversaturated. The fluorescence of minor band 1 in lanes 1 and 2 is ~4% of the fluorescence of band 2 in lane 1. Arrows labelled with 1-3 in panel B correspond to the M88 protomers with 1-3 residues modified by Alexa Fluor 633, respectively.


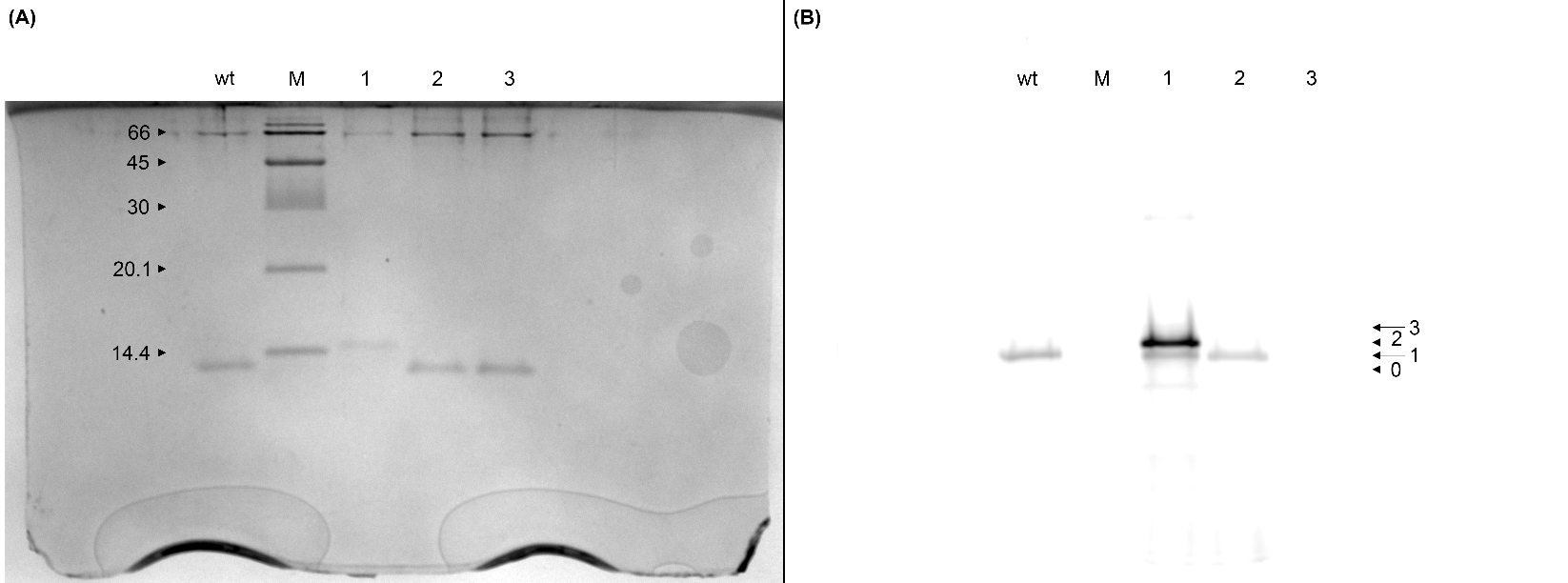


**Supplementary Figure S2: Coomassie (A) and fluorescent (B) gel images visualizing the reactions of both wild-type streptavidin (wt) and M88 (1-3) with Alexa Fluor 633 maleimide**. Lanes 1-3 correspond to the same reaction conditions as Fig 2 and Fig S1. Wild-type streptavidin, which contains no Cys, can also react with the maleimide label, indicating a much less efficient side-reaction likely involving amino groups. Arrows labelled with 1-3 in panel B correspond to the M88 protomers with 1-3 residues modified by Alexa Fluor 633, respectively, as described for Fig. S1.s


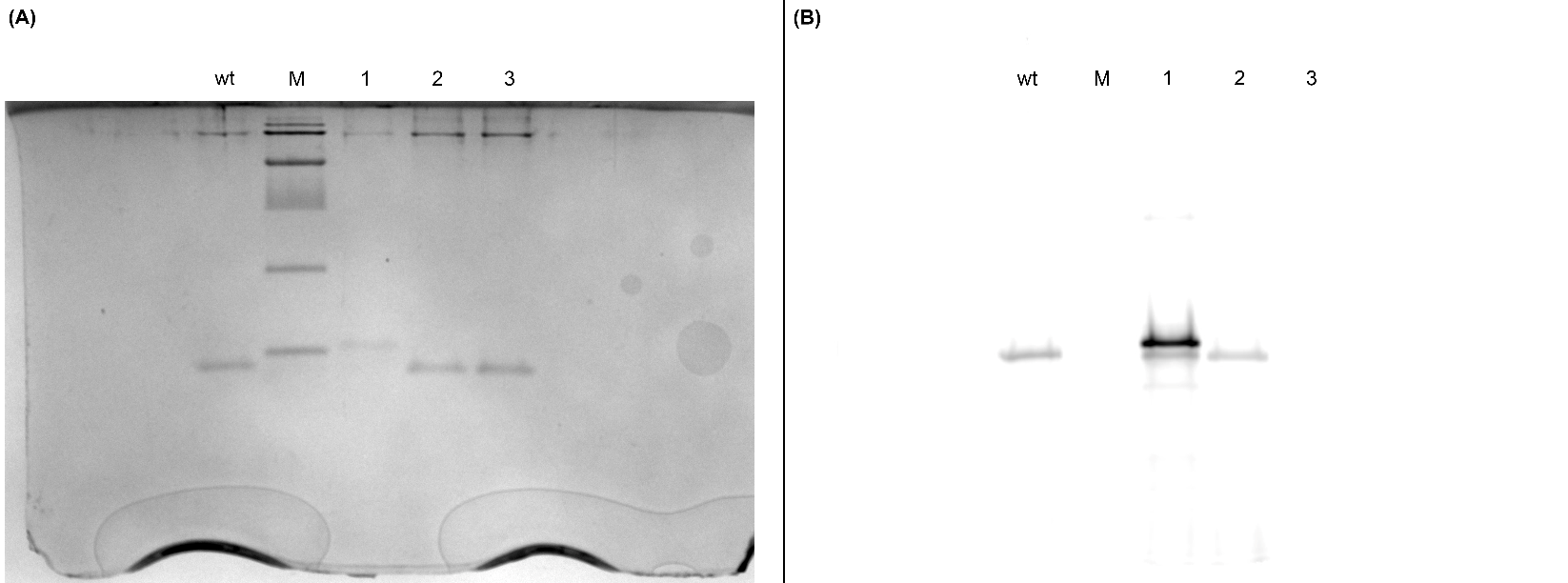


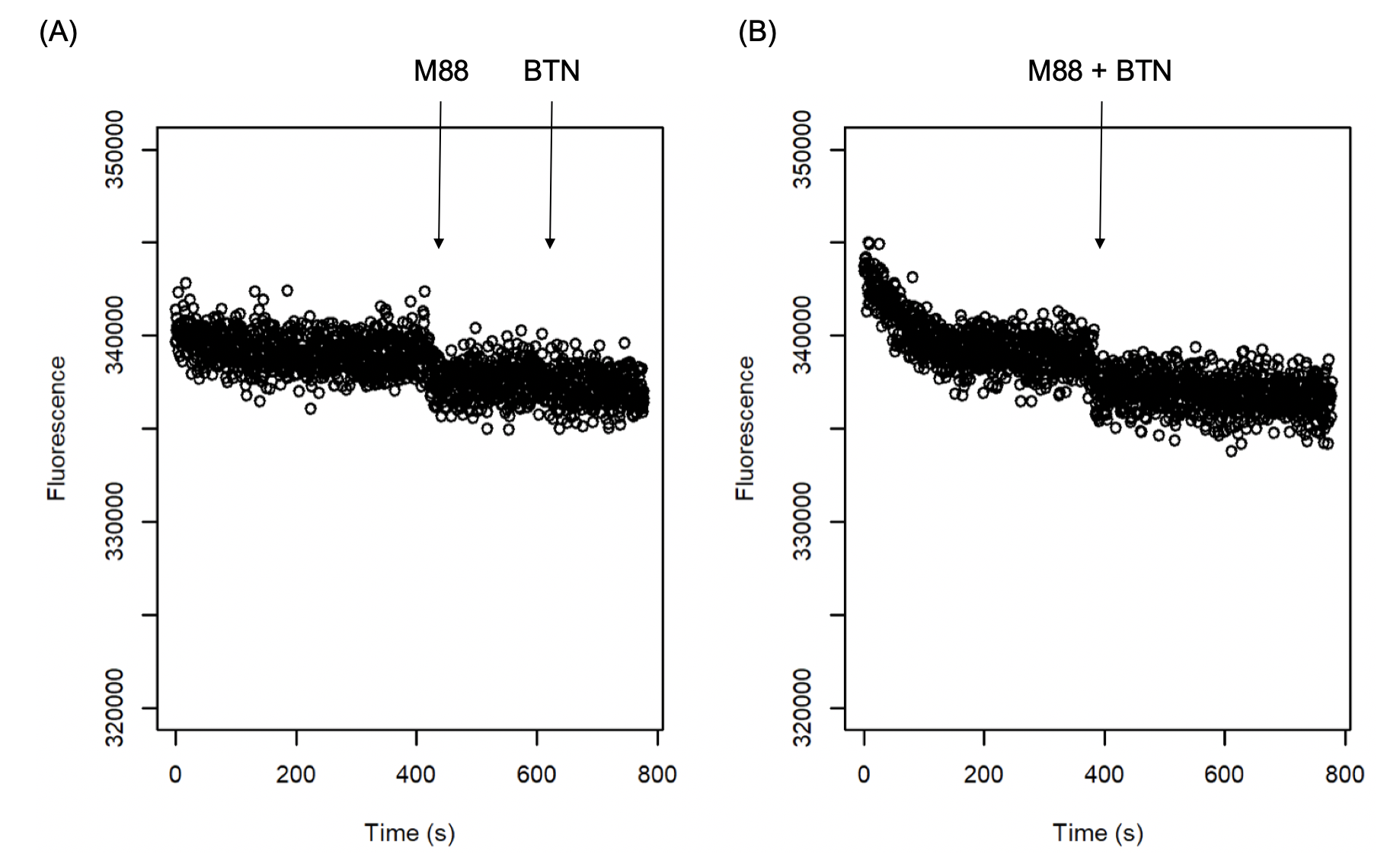


**Supplementary Figure S3: Binding of fluorescein to reduced M88 at 25°C.** (A) 10 nM fluorescein was equilibrated at 21°C. 50 nM M88 was added at approximately 400 s and 0.4% decrease in fluorescence was observed. This is only slightly higher than the 0.2% decrease expected from dilution of the solution when M88 is added and is insignificant compare to the quenching observed when B4F binds M88. At 600 s, biotin was added but it had little detectable effect on fluorescence. (B) M88 pre-bound to biotin was added to fluorescein. The small decrease in fluorescence is similar to that seen in panel (A) and can be attributed to dilution and a small amount of nonspecific binding that is much smaller than the quenching seen with B4F.


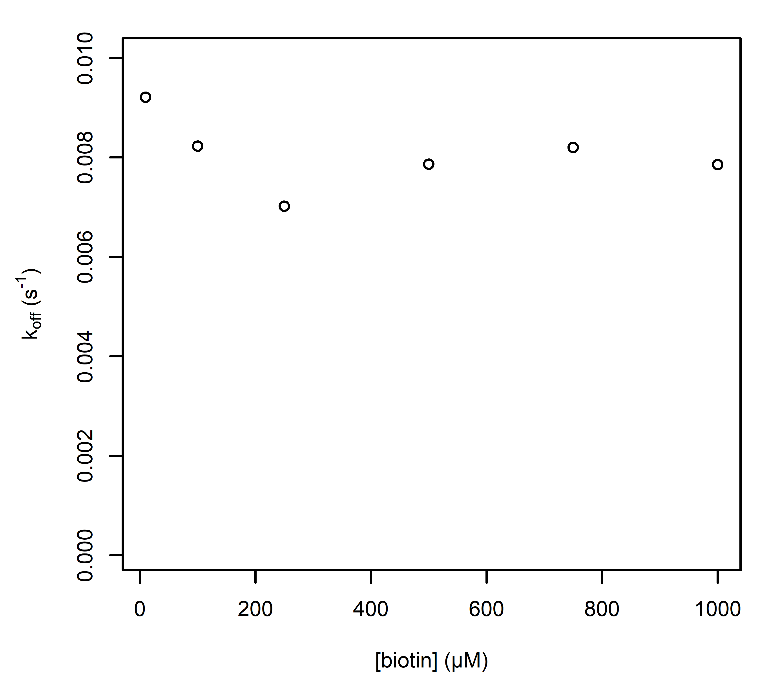


**Supplementary Figure S4: Off-rate constants for the dissociation of B4F from reduced M88 at 60°C using different concentrations of competing unlabelled biotin.** B4F (10 nM) was added to an excess of M88 (50 nM), binding to which causes fluorescence quenching. An excess of unlabelled biotin was added to prevent re-association of B4F and the increase in fluorescence was monitored over time. The apparent rates of dissociation for B4F are constant over the range of 0.1-1.0 mM biotin, confirming the lack of rebinding or secondary effects due to the excess amount of free biotin. At biotin concentrations at or below 10 μM, the rebinding of B4F to M88 significantly alters the apparent rate and amplitude of the change in fluorescence.
